# Adiponectin is required for maintaining normal body temperature in a cold environment

**DOI:** 10.1186/s12899-017-0034-7

**Published:** 2017-10-23

**Authors:** Qiong Wei, Jong Han Lee, Hongying Wang, Odelia Y. N. Bongmba, Chia-Shan Wu, Geetali Pradhan, Zilin Sun, Lindsey Chew, Mandeep Bajaj, Lawrence Chan, Robert S. Chapkin, Miao-Hsueh Chen, Yuxiang Sun

**Affiliations:** 1 USDA/ARS Children’s Nutrition Research Center, Department of Pediatrics, Baylor College of Medicine, Houston, TX 77030 USA; 2 Division of Endocrinology, Zhongda hospital, Southeast University, Nanjing, Jiangsu Province People’s Republic of China 210002; 3 College of Pharmacy, Gachon University, Incheon, 21936 South Korea; 4 Department of Nutrition and Food Science, Texas A&M University, 214D Cater-Mattil; 2253 TAMU, College Station, TX 77843 USA; 5 Laboratory of Lipid & Glucose Metabolism, The First Affiliated Hospital of Chongqing Medical University, Chongqing, Sichuan province People’s Republic of China 400016; 6 Institute of Biosciences and Technology, Houston, TX 77030 USA; 7 Department of Medicine, Baylor College of Medicine, Houston, TX 77030 USA; 8 Huffington Center on Aging, Baylor College of Medicine, Houston, TX USA

**Keywords:** Adiponectin, Thermogenesis, Brown adipose tissue, Beige cells, Cold exposure

## Abstract

**Background:**

Thermogenic impairment promotes obesity and insulin resistance. Adiponectin is an important regulator of energy homeostasis. While many beneficial metabolic effects of adiponectin resemble that of activated thermogenesis, the role of adiponectin in thermogenesis is not clear. In this study, we investigated the role of adiponectin in thermogenesis using adiponectin-null mice (*Adipoq* ^−/−^).

**Methods:**

Body composition was measured using EchoMRI. Metabolic parameters were determined by indirect calorimetry. Insulin sensitivity was evaluated by glucose- and insulin- tolerance tests. Core body temperature was measured by a TH-8 temperature monitoring system. Gene expression was assessed by real-time PCR and protein levels were analyzed by Western blotting and immunohistochemistry. The mitochondrial density of brown adipose tissue was quantified by calculating the ratio of mtDNA:total nuclear DNA.

**Results:**

Under normal housing temperature of 24 °C and ad libitum feeding condition, the body weight, body composition, and metabolic profile of *Adipoq* ^−/−^ mice were unchanged. Under fasting condition, *Adipoq* ^−/−^ mice exhibited reduced energy expenditure. Conversely, under cold exposure*, Adipoq* ^−/−^ mice exhibited reduced body temperature, and the expression of thermogenic regulatory genes was significantly reduced in brown adipose tissue (BAT) and subcutaneous white adipose tissue (WAT). Moreover, we observed that mitochondrial content was reduced in BAT and subcutaneous WAT, and the expression of mitochondrial fusion genes was decreased in BAT of *Adipoq* ^−/−^ mice, suggesting that adiponectin ablation diminishes mitochondrial biogenesis and altered mitochondrial dynamics. Our study further revealed that adiponectin deletion suppresses adrenergic activation, and down-regulates β3-adrenergic receptor, insulin signaling, and the AMPK-SIRT1 pathway in BAT.

**Conclusions:**

Our findings demonstrate that adiponectin is an essential regulator of thermogenesis, and adiponectin is required for maintaining body temperature under cold exposure.

**Electronic supplementary material:**

The online version of this article (10.1186/s12899-017-0034-7) contains supplementary material, which is available to authorized users.

## Background

Adiponectin is a 30 kDa protein hormone secreted by adipocytes; it has high concentrations (0.5–30 mg/ml) in the circulation, and displays a wide range of metabolically beneficial effects [1–7]. Several lines of evidence show that adiponectin is involved in lipid metabolism [3, 4]. In insulin-resistant mouse models, adiponectin treatment improves insulin sensitivity [5, 6]. Clinical studies also reveal a correlation between circulating adiponectin and insulin sensitivity; and adiponectin levels are lower in obese humans compared to normal-weight subjects [1, 6, 7]. These findings suggest that adiponectin might be a promising candidate for prevention/treatment of metabolic syndrome and insulin resistance.

There are two types of adipose tissues: energy-storing white adipose tissue (WAT) and energy-burning brown adipose tissue (BAT). WAT is the main organ for long-term energy storage in mammals. In WAT, lipids are stored as triglycerides under positive energy balance, and fatty acids are released as metabolic fuel under negative energy balance [8, 9]. In contrast, BAT is primarily responsible for non-shivering thermogenesis, which converts fat into heat. Thermogenesis is vital in maintaining normal body temperature, which is crucial for the cellular functions of all cells in the body. BAT is a key protective mechanism for preventing hypothermia [10, 11]. In addition to BAT, there are brown-like adipocytes in subcutaneous WAT, aka brite/beige cells, which also possess thermogenic properties [8, 11, 12]. Emerging evidence reveals that non-shivering thermogenesis is linked to energy expenditure in healthy adult humans [13–16], and thermogenic activation improves glucose homeostasis [17–20]. Uncoupling protein 1 (UCP-1) is a key mitochondrial regulator of thermogenesis. Upon activation, UCP-1 dissipates the transmembrane proton gradient to generate heat [21]. It is important to note that the UCP-1 ablation in mice failed to display an obesogenic phenotype under normal laboratory housing temperature (18–22 °C), but animals became obese under thermoneutral temperature (30 °C) when thermal stress is eliminated [22]. This new revelation indicates that housing temperature has profound effects on metabolism.

It has been reported that cold exposure increases adiponectin levels [23, 24]. Acute cold exposure in rodents activates both non-shivering and shivering thermogenesis by activating the sympathetic nervous system (SNS) [25]. Cold exposure increases UCP-1 expression, as well as UCP-1 activity in BAT and subcutaneous WAT [26]. Although many beneficial metabolic effects of adiponectin phenocopy that of thermogenic activation [1–7], the effect of adiponectin on thermogenesis remains controversial. Two recent studies show opposite effects of adiponectin on thermogenesis [27, 28]. In the current study, we investigated the effects of adiponectin on thermogenic regulation using *Adipoq* ^−/−^ mice, which have normal body weight and insulin sensitivity [29]. Initial characterization of these *Adipoq* ^*−/−*^ mice showed minimal phenotype under unchallenged conditions [29]; subsequent studies revealed these mice have hepatic steatosis and mitochondrial dysfunction, and are prone to liver injuries [30]. Insulin levels have profound effects on thermogenesis [31–34], and thermogenic impairment has been shown to be associated with insulin resistance [35]. This line of *Adipoq* ^−/−^ mice with normal insulin sensitivity would allow us to investigate the metabolic effects of adiponectin independent from body weight and insulin action. In the current study, we used this *Adipoq* ^*−/−*^ mouse line to investigate the thermogenic effect of adiponectin.

We studied the core body temperature and expression of the genes involved in thermogenesis and mitochondrial dynamics in BAT and subcutaneous WAT under conditions of normal housing temperature (24 °C), negative energy balance (24 h fasting), and 4 °C cold exposure. Our results indicate that adiponectin plays an important role in thermoregulation. We found that adiponectin is required for sustaining body temperature under energy-deficient and cold challenged conditions, also adiponectin activates thermogenesis to enhance lipid metabolism to protect against hypothermia.

## Methods

### Animals

The *Adipoq* ^−/−^ mice were previously reported; they have been backcrossed to C57BL/6 J for at least 10 generations (with >99% C57BL/6 J background) [29]. Age-matched littermate male WT (*n* = 6–10) and *Adipoq* ^−/−^ (*n* = 6–10) mice were used in the studies. Animals were housed under normal housing temperature (24 ± 1 °C) with 12 h light/dark cycle (6 AM to 6 PM), and given free access to chow and water. All experiments were approved by the Institutional Animal Care and Use Committee (IACUC) of Baylor College of Medicine.

### Body composition and indirect calorimetry

Whole-body composition (fat and lean mass) of mice was measured by an Echo MRI-100 whole-body composition analyzer (Echo Medical Systems, Houston, TX), following manufacturer’s instructions as previously described [36]. Metabolic parameters were obtained by using Comprehensive Laboratory Animal Monitoring System (CLAMS) of Columbus Instruments (Columbus, OH). To minimize the confounding effects of stress, mice (6 WT, 6 *Adipoq* ^−/−^) were individually caged for 1 week and then placed in metabolic cages for at least 4 days prior to the indirect calorimetry testing. After 24 h of acclimatization in the calorimetry chambers, indirect calorimetry data were collected for 48 h. Energy expenditure was normalized to lean body mass [37]. Locomotor activity was measured using infrared beams to count the number of beam breaks during the recording period. Resting metabolic rate (RMR) was determined on the final day in the metabolic cages. Mice were fasted from 6 AM when the light was on, and RMR was calculated using the three lowest energy expenditure data points between 10 am and 2 pm as we previously described [36, 38].

### Insulin tolerance test (ITT) and glucose tolerance test (GTT)

The insulin tolerance tests (ITT) were carried out with WT (*n* = 6) and *Adipoq* ^*−/−*^ (*n* = 7) male mice. Blood glucose concentration was measured using OneTouch Ultra blood glucose meter and test strips (LifeScan). For ITT, mice were *i.p.* injected with human insulin (Eli Lilly) at a dose of 1.0 U/kg of body weight following a 6 h of fasting period in the morning. Blood glucose was assessed at 0, 30, 60, 90 and 120 min after injections. For glucose tolerance tests (GTT), mice were *i.p.* injected with glucose at a dose of 2.0 g/kg body weight following a 18 h overnight fasting. The blood glucose was measured at 0, 15, 30, 60 and 120 min after injection.

### Core body temperature measurement

To investigate the effects of acute cold exposure on body temperature, mice (10 WT, 9 *Adipoq* ^−/−^) were caged individually in a 4 °C cold room for 6 h with free access to food and water. Rectal temperature of the mice was measured using a TH-8 temperature monitoring system (Physitemp, Clifton, NJ). The probe was lubricated with petroleum jelly and then gently inserted into the rectum of the mice to a depth of approximately 1.5–2 cm, stabilized temperature was subsequently recorded.

### Quantification of mitochondrial density of BAT

The mitochondrial density was assessed as we previously described [39]. Briefly, fresh interscapular BAT was homogenized in 1 mL isolation buffer (300 mM sucrose, 1 mM EDTA, 5 mM MOPS, 5 Mm KH_2_PO_4_, 0.01% BSA, pH 7.4), centrifuged at 800 g for 10 min at 4 °C, and then the pellet of nuclei was saved. The supernatant was subsequently further centrifuged at 8000 g for 10 min at 4 °C, with the resulting pellet saved as the mitochondrial fraction. Nuclear DNA was extracted using the standard phenol/chloroform method. PCR was performed to amplify the 162-nt region of the mitochondrial NADH dehydrogenase subunit 4 genes. The PCR product was purified using the high-pure PCR template preparation kit (Roche, Indianapolis, IN). The nuclear DNA and the amplified PCR products were quantified using a NanoDrop spectrophotometer (ND-1000 Thermo Scientific, Waltham, MA). The ratio of mtDNA:total DNA was then calculated.

### Real-time PCR

Total RNA of tissues was isolated using TRIzol Reagent (Invitrogen, Carlsbad, CA). RNA was treated with DNase and run on agarose gels to validate RNA quality. The cDNA was synthesized from 500 ng RNA using the SuperScript III First-Strand Synthesis System (Invitrogen, Carlsbad, CA). Real-time PCR was performed on a Bio-Rad q-PCR machine using the SYBR Green PCR Master Mix, according to the protocol provided by the manufacturer. The expression was normalized by 18 s. Primers of genes used are available upon request.

### Western blot analysis

Tissue was sonicated in 1X RIPA Buffer (20 mM Tris-HCl [pH 7.5], 150 mM NaCl, 1 mM Na_2_EDTA, 1 mM EGTA, 1% NP-40, 1% sodium deoxycholate, 2.5 mM sodium pyrophosphate, 1 mM b-glycerophosphate, 1 mM Na_3_VO_4_,1 μg/ml leupeptin) containing complete Phosphatase Inhibitor Cocktail (PhosSTOP) and Protease Inhibitor Cocktail (Roche Inc.). Protein concentrations were determined using BCA (bicinchoninic acid) Protein Assay kit (Pierce, Rockford, IL). Protein (20 μg) was separated and transferred to a polyvinylidene difluoride membrane. Membranes were blocked in Tris-buffered saline with TWEEN® 20 (TBS-T, 50 mM Tris-HCl [pH 7.5–8.0], 150 mM NaCl, and 0.1% Tween 20) in 5% non-fat milk for 1 h at room temperature, and incubated overnight at 4 °C with phosphorylated and total AMPK (p-AMPK and t-AMPK) from Cell Signaling Technology (1:1000 in 3% BSA). Pierce ECL Western Blotting Substrate was used to detect the specific proteins. Densitometry analyses were performed using NIH ImageJ software.

### Immunohistochemistry

Immunohistochemistry was performed as described [40, 41]. Briefly, tissue slides of BAT and inguinal fat were dewaxed in xylene, rehydrated in ethanol (100%, then 95%, ethanol washes) and rinsed in PBS. A heat-induced antigen retrieval step with Citric Acid Based Antigen Unmasking Solution (Vector laboratories, Burlingame, CA) was used to unmask antigens. To block endogenous peroxidases, slides were incubated in 3% H_2_O_2_ for 30 min at room temperature and then rinsed in PBS. Before primary antibody was applied, slides were soaked in blocking solution (containing 5% sheep serum, 0.2% BSA, and 0.1% Triton X-100 in PBS) for 1 h at room temperature. The following antibodies were used: rabbit-anti UCP-1 (1:50; Abcam) and mouse- anti mitochondria (1:25; Abcam). All antibody staining was performed at 4 °C overnight, followed by incubation with 1:1000 diluted anti-biotin secondary antibody (Vector Laboratories) for 45 min at room temperature. Slides were developed using a DAB kit (Vector Laboratories) and imaged using a DS-Fi1 camera connected to a Nikon E80i stereomicroscope. Images were processed using Nikon imaging software, NIS Elements RA3.2.

### Data analysis

Statistical analysis was performed using Two-way ANOVA or one-way ANOVA followed by Tukey’s post-hoc analysis. Data were presented as Mean ± SEM; *P* < 0.05 considered statistically significant.

## Results

### Body composition and insulin sensitivity

**Fig. 1:**
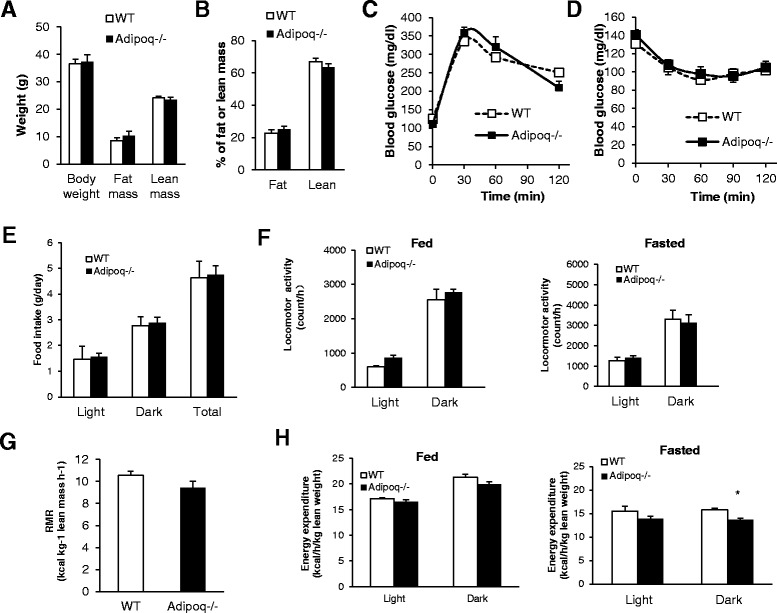
*Adipoq* ^−/−^ mice show similar body composition, insulin sensitivity and metabolic profile under normal housing conditions. **a** Body weight, fat and lean mass. **b** Body composition of fat and lean. **c, d** Glucose tolerance tests (GTT) and Insulin tolerance tests (ITT) at 6-months of age. Calorimetry analysis of 5-month old male WT and *Adipoq* ^−/−^ mice: (**e**) Daily food intake, (**f**) Physical activity under *ad. lib*-fed and fasted conditions. **g** Resting metabolic rate (RMR) normalized by lean mass, and (**h**) Energy expenditure normalized by lean mass under *ad. lib*-fed and fasted conditions. *n* = 6–7.*P < 0.05, WT vs. *Adipoq* ^*−*/−^ mice

The body weights of *Adipoq* ^−/−^ mice were not different from that of their WT controls (Fig. 1a). There was a slight trend of increase of total fat mass in *Adipoq* ^−/−^ mice, but it did not reach statistical significance (Fig. 1b). *Adipoq* ^−/−^ mice had normal fasting blood glucose and insulin levels (data not shown). There was no difference in glucose response during GTT (Fig. 1c). Similarly, there was no difference detected in ITT (Fig. 1d).

### Metabolic characterization

We next examined the metabolic profiles of *Adipoq* ^−/−^ mice using Comprehensive Laboratory Animal Monitoring System (CLAMS). Our data showed no difference in total daily food consumed by *Adipoq* ^−/−^ mice compared with WT mice (Fig. 1e), indicating that adiponectin ablation has no effect on total daily energy intake. To further assess whether there were difference in physical activity, we analyzed spontaneous locomotor activity. The locomotor activity was not changed under either ad lib. Fed or fasted conditions (Fig. 1f). The resting metabolic rate (RMR) was similar between WT and *Adipoq* ^−/−^ mice (Fig. 1g), which implies that the basal metabolic rate was unaffected. While *Adipoq* ^−/−^ mice exhibited no difference in energy expenditure under normal ad lib. Fed condition (Fig. 1h), the mice showed significantly reduced energy expenditure under fasting condition during the dark cycle (Fig. 1h).

### Core body temperature during cold exposure

**Fig. 2:**
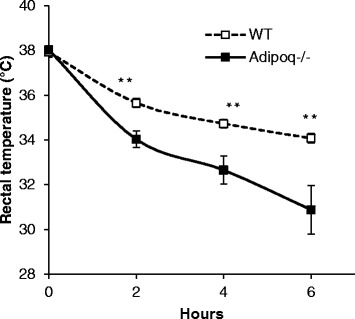
*Adipoq* ^−/−^ mice are sensitive to cold exposure**.** Ten-month old male mice were individually caged at 4 °C and provide with free access to food and water. Rectal temperature was recorded every 2 h. *n* = 9–10. **P < 0.001, WT vs. *Adipoq* ^*−*/−^ mice

To assess the thermogenic phenotype of *Adipoq* ^−/−^ mice, mice were challenged with 4 °C cold exposure for 6 h. We measured the rectal temperature (as readout of core body temperature) every 2 h during the cold exposure. The rectal temperature of *Adipoq* ^−/−^ mice was significantly lower than that of WT mice during cold exposure, and the difference became more pronounced under prolonged cold exposure, i.e., the temperature difference between WT and *Adipoq* ^−/−^ mice increased from 0.03 °C at 0 h to 3.70 °C after 6 h of cold exposure (Fig. 2).

### Expression of thermogenic genes in BAT in response to cold challenge

**Fig. 3:**
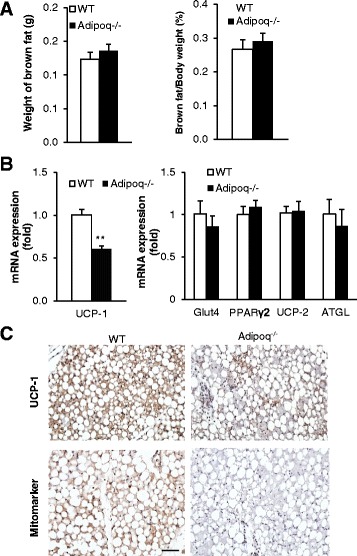
Adiponectin ablation reduces BAT thermogenic activity**.** BAT from 10-month old WT and *Adipoq* ^−/−^ mice was collected immediately following a 6 h cold (4 °C) exposure. **a** BAT weight and BAT percentage (compared to body weight). **b** Expression of thermogenic genes. **c** BAT immunohistochemical images of UCP-1 and Mitomarker. Brown color represents specific staining for UCP-1 or Mitomarker. Scale bar is 50 μm. *n* = 6. **P < 0.001, WT *v.s* A*dipoq* ^*−*/−^ mice

To understand the underlying molecular mechanisms of adiponectin-mediated thermogenesis, BAT was collected from WT and *Adipoq* ^−/−^ mice immediately following a 6 h cold exposure. There was no difference in total BAT weight or BAT/body weight ratio between WT and *Adipoq* ^−/−^ mice (Fig. 3a). The expression of thermogenic regulator UCP-1 was significantly decreased in BAT of *Adipoq* ^−/−^ mice compared to WT mice, while the expression of glucose uptake regulator (Glut4), adipogenic regulator (PPARγ2), fat utilization regulator (UCP-2), and lipolytic enzyme (ATGL) was unchanged (Fig. 3b). We also detected reduced UCP1 protein in BAT of *Adipoq* ^−/−^ mice (Additional file 1: Figure S1). Consistently, immunohistochemistry analysis showed that the immunostainings of UCP-1 and Mitomarker were lower in BAT of *Adipoq* ^−/−^ mice compared to WT mice (Fig. 3c). The reduced body temperature in *Adipoq* ^−/−^ mice under cold exposure indicates that adiponectin deficiency suppresses BAT thermogenesis.

**Fig. 4:**
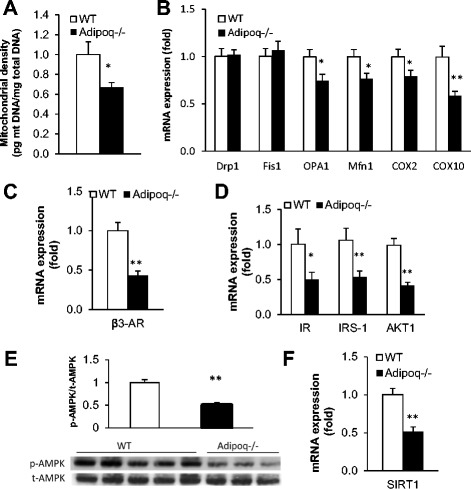
Adiponectin ablation reduces the thermogenic capacity of mitochondria in BAT. BAT from male 10-month old WT and *Adipoq* ^−/−^ mice were collected after a 6 h of 4 °C cold exposure. **a** Quantification of mitochondrial density. **b** Expression of mitochondrial dynamic genes. **c, d** Expression of putative adiponectin-mediated thermogenic regulators. **e** Representative Western blots of AMPK activation in BAT from WT and *Adipoq* ^−/−^ mice. p-AMPK for phosphorylated AMPA; t-AMPK for total AMPK. **f** Expression of SIRT1, a downstream target of AMPK. *n* = 6. *P < 0.05, **P < 0.001 WT vs. A*dipoq* ^*−*/−^ mice

Mitochondrial function is determined by mitochondrial biogenesis and mitochondrial dynamics [42]. To determine whether adiponectin deletion affected mitochondrial biogenesis in BAT, we analyzed mitochondrial density by measuring the ratio of mitochondrial DNA:nuclear DNA. Mitochondrial density of BAT from *Adipoq* ^−/−^ mice was lower than that of WT mice (Fig. 4a), indicative of reduced mitochondrial biogenesis. Mitochondrial dynamics is another crucial component that controls mitochondrial function and cell survival [43–45]. Mitochondrial dynamics consist of the processes of the ‘joining event’ of fusion and the ‘dividing event’ of fission; the balance between fusion and fission is essential for the maintenance of normal mitochondrial function [44]. Fusion is mediated by mitofusins (Mfns) and optic atrophy gene 1 (OPA1); fission is mediated by dynamin-related protein 1 (Drp1) and fission 1 (Fis1) protein. To study mitochondrial dynamics of BAT, we analyzed key regulatory genes for mitochondrial dynamics and subunits of mitochondrial respiratory chain complexes. While the expression of mitochondrial fission genes (Drp1 and Fis1) in BAT of *Adipoq* ^−/−^ mice was unchanged, the expression of mitochondrial fusion genes (OPA1 and Mfns) was significantly decreased (Fig. 4b). Consistent with the decreased thermogenic function, the expression of the subunits of mitochondrial respiratory chain complexes IV (COX-2 and COX10) were decreased in *Adipoq* ^−/−^ mice (Fig. 4b).

### Potential regulators and signaling pathways mediating the thermogenic effect of adiponectin

To further study the molecular mechanisms mediating thermogenic impairment induced by adiponectin deficiency, we investigated the expression of β3-adrenergic receptor (β3-AR). β3-AR expression was decreased in BAT of *Adipoq* ^−/−^ mice (Fig. 4c), suggesting reduced adrenergic activation. Insulin signaling in BAT is activated by cold stress [46] and insulin signaling mediator AKT (also known as “protein kinase B”, PKB) has been linked to mitochondrial biogenesis [47, 48]. We assessed insulin signaling in BAT by studying the expression of key regulators of insulin signaling: insulin receptor (IR), insulin receptor substrate 1 (IRS-1) and AKT. In line with the reduced thermogenic phenotype, the expression of IR, IRS-1 and AKT1 in BAT of *Adipoq* ^−/−^ mice were significantly decreased in BAT of *Adipoq* ^−/−^ mice (Fig. 4d). We also detected reduced IRS1 protein in BAT of *Adipoq* ^−/−^ mice (Additional file 1: Figure S1). Adiponectin, AMP-activated protein kinase (AMPK) and sirtuin 1 (SIRT1) signaling have been suggested to promote mitochondrial biogenesis in muscle [49–51]. To determine whether AMPK and SIRT1 mediate the mitochondrial effect of adiponectin on BAT, we examined the activity of AMPK and the expression of SIRT1. Adiponectin ablation decreased phosphorylated AMPK (Fig. 4e) and down-regulated SIRT1 (Fig. 4f) in BAT of *Adipoq* ^−/−^ mice.

### The expression of thermogenic genes in subcutaneous WAT in response to cold stress

**Fig. 5:**
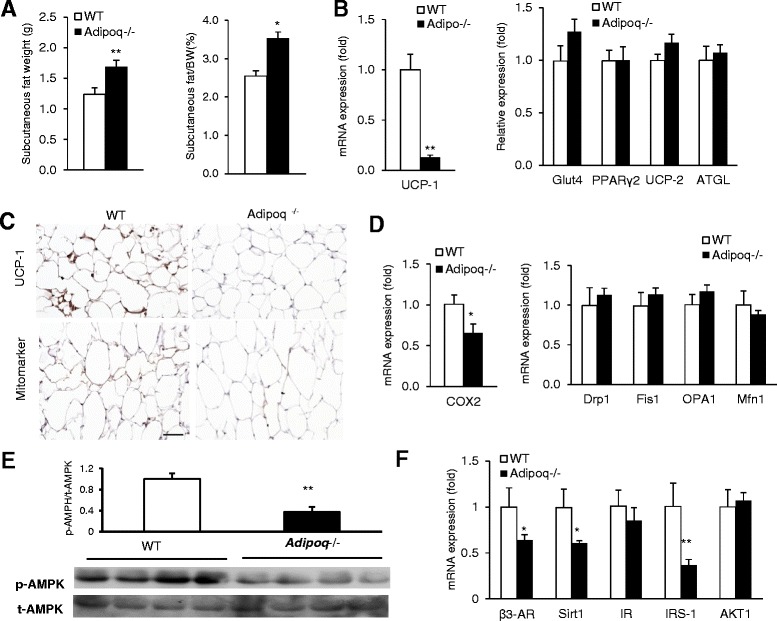
Adiponectin ablation reduces thermogenic activity in subcutaneous fat. Subcutaneous (inguinal) fat from10-month old WT and *Adipoq* ^*−*/−^ mice was collected after 6 h following 4 °C cold exposure. **a**Subcutaneous fat weight and percentage ratio compared to body weight (BW). **b** Expression of thermogenic genes. **c** Immunohistochemical images of the UCP-1 and Mitomarker in inguinal fat. Scale bar is 50 μm. **d** Expression of mitochondrial dynamic genes. **e** Representative Western blots of AMPK activation in inguinal fat of WT and *Adipoq* ^−/−^ mice. p-AMPK for phosphorylated AMPA; t-AMPK for total AMPK. **f** Expression of regulators potentially involved in adiponectin-mediated thermogenesis. *n* = 6. *P < 0.05, **P < 0.001 WT vs. *Adipoq* ^*−*/−^

“Beige” adipocytes in subcutaneous WAT have been shown to possess thermogenic activity similar to classic BAT [11]. The weight ratio of subcutaneous WAT to total body weight was significantly higher in *Adipoq* ^−/−^ mice than WT mice (Fig. 5a). Similar to that of BAT, UCP-1 expression in inguinal fat was decreased in cold-challenged *Adipoq* ^−/−^ mice, while the expression of Glut4, PPARγ2, UCP2 and ATGL were unchanged (Fig. 5b **).** Consistently, immunohistochemical analysis showed that UCP-1 and Mitomarker were significantly lower in WAT of *Adipoq* ^−/−^ mice than that of WT mice (Fig. 5c). However, unlike BAT, the mitochondrial dynamic genes (OPA1, Mfns, COX-2) were unchanged, while the expression of the mitochondrial respiratory chain complexes IV gene (COX-2) was decreased (Fig. 5d). Similar to BAT, adiponectin ablation decreased phosphorylated AMPK (Fig. 5e) and the expression of adrenergic receptor β3-AR and SIRT1 in subcutaneous (inguinal) fat in *Adipoq* ^−/−^ mice (Fig. 5f).

## Discussion

Non-shivering thermogenesis in BAT and “beige” adipocytes in subcutaneous WAT dissipates energy as heat to protect against hypothermia, that may protect against obesity [15]. Our findings reveal that adiponectin ablation exacerbates thermogenic dysfunction under cold stress, exhibiting reduced thermogenesis and impaired ability to maintain body temperature. Our study indicates that adiponectin plays a crucial role in thermogenesis under fasting and cold stress conditions. Specifically, our data show that adiponectin-ablated mice have normal thermoregulation at regular housing temperature and ad lib. Fed condition, but exhibit thermogenic impairment under fasting and cold condition. Thus, adiponectin is not required for thermogenic regulation under normal feeding and housing environment, but is required for heat production and body temperature maintenance under negative energy balance and cold exposure. Our data show that the rectal temperature of *Adipoq* ^−/−^ mice was significantly lower than that of WT mice under acute cold exposure, which decreased drastically with the length of cold exposure. It has been reported that cold exposure (4 °C for 12–24 h) reduces serum adiponectin in mice [52]. In accordance with our findings, another group recently reported that chronic cold exposure induces adiponectin accumulation in visceral fat, which in turn stimulates thermogenic activation [27]. A recent report has shown that beige adipose tissue fails to produce appreciable thermogenic activation in response to chronic cold [53]. So even though changes in thermogenic genes and protein were detected in both BAT and subWAT of adiponectin null mice, the thermogenic phenotype we observed is likely primarily due to BAT. Opposite from our finding, using a different adiponectin knockout mouse line with severe insulin resistance, Qiao et al. reported that adiponectin deficiency increases thermogenesis under cold stress [28]. It is known that insulin level and insulin sensitivity affects thermogenesis [32–34]*.* We intentionally used the *Adipoq*−/− mouse line with no insulin resistance in our study. The different thermogenic phenotypes observed in these 2 different adiponectin-null models could be due to differences in insulin sensitivity status. In addition, study by Qiao et al. was conducted in young mice (2 months old); our study was conducted in older mice (10 months of age). It is know there is significant thermogenic decline in aging, and adiponectin level is positively correlated with aging [54]. Thus, the age difference may also contribute to the differential thermogenic phenotypes. Our findings collectively suggest that adiponectin is responsive to both acute and chronic cold insults, it is required to maintain body temperature under cold exposure, and it may be used to treat hypothermia. It is well documented that adiponectin has many beneficial metabolic effects, many of which phenocopy that of thermogenic activation [1–7]. Our finding that adiponectin deletion impairs thermogenesis is in agreement with the thermo-protective effect of exogenous adiponectin. Thermogenesis is affected not only by the cold, it is also affected by food intake (thermic effect of food). Also, the normal animal housing condition temperature (24 °C) is lower than the thermoneutral temperature (~30 °C) for mice [55]. For future studies, it would be interesting to study the fasted mice under thermoneutrality.

UCP-1 is a key regulator of thermogenesis, which allows protons to enter the mitochondrial matrix and dissipate energy as heat [9]. UCP-1 is the hallmark regulator which mediates cold-induced non-shivering thermogenesis [56]. We detected decreased UCP-1 mRNA expression in BAT and subcutaneous fat of *Adipoq* ^−/−^ mice under cold exposure, indicating that adiponectin-mediated thermoregulation is mediated through UCP-1. Thermogenic function of brown/beige adipocytes is determined by content and functional capacity of mitochondria [44]. While mitochondrial content is determined by mitochondrial biogenesis, mitochondrial function is controlled by mitochondrial structure integrity and dynamics. We detected a lower mitochondrial DNA content ratio in the BAT of *Adipoq* ^−/−^ mice after cold exposure, implying reduced mitochondrial biogenesis. Several studies show abnormal mitochondrial structure in obese and type 2 diabetic patients [57, 58]. Interestingly, while expression of mitochondrial fusion genes was decreased in the BAT of *Adipoq* ^−/−^ mice, the expression of fission genes were unchanged. The imbalance between fusion and fission in the BAT of *Adipoq* ^−/−^ mice can lead to mitochondrial fragmentation, and the impairment of mitochondrial dynamics can lead to mitochondrial dysfunction. In agreement with the reduced mitochondrial biogenesis and impaired mitochondrial dynamics, the expression of mitochondrial subunit genes of mitochondrial respiratory chain complexes IV (COX-2 and COX10) was decreased in BAT of *Adipoq* ^−/−^ mice, further supporting reduced thermogenic capacity [30]. COX-2 is known to be encoded by mtDNA [59]. We observed reduced expression of COX-2 in BAT of *Adipoq* ^−/−^ mice, which is consistent with the reduced mitochondrial DNA detected in BAT of *Adipoq* ^−/−^ mice, supporting reduced mitochondrial biogenesis. Our data demonstrate that Adiponectin deficiency decreases mitochondrial biogenesis and impairs dynamics, thereby attenuating thermogenic activity.

Activation of adrenergic signaling via β3-adrenergic receptor (β3-AR) is essential for thermogenic activation in brown adipocytes [52]. It has been reported that peripheral administration of adiponectin increases β3-AR expression in BAT of mice and rectal temperature [60]. We found that β3-AR is decreased in BAT and subcutaneous WAT of *Adipoq* ^−/−^ mice after cold exposure. Thus, the thermogenic phenotype of *Adipoq* ^−/−^ mice is likely linked to β-adrenergic activation. The β-adrenergic signaling in brown adipocytes can be activated centrally via sympathetic nerve activity (SNA), or peripherally by circulating adiponectins. Masaki et al. reported that peripheral, not central, adiponectin administration increases SNA and UCP1 expression in the BAT, and elevates rectal temperature [61]. Hui et al. recently reported that adiponectin directly induces browning of subcutaneous adipose tissues by promoting M2 macrophage proliferation [27]. Thus, we believe that the phenotype we see in adiponectin KO is primarily taken place at the adipocyte level. Thermogenic defects are associated with insulin resistance in BAT [35], and cold stress has been shown to stimulate the insulin-signaling pathway in BAT to improve glucose homeostasis and insulin sensitivity [32, 62]. Our data show that the insulin signaling in BAT was suppressed in *Adipoq* ^−/−^ mice after cold exposure, which suggests that adiponectin deficiency impairs the insulin sensitivity of brown adipocytes, which may lead to the suppression of thermogenic activity.

**Fig. 6:**
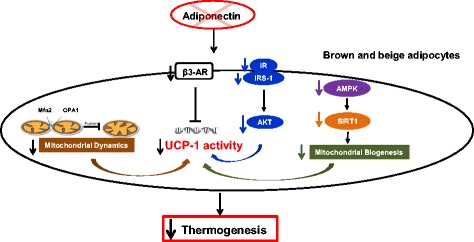
A Schematic diagram of adiponectin-mediated thermogenic regulation in *brown* and “*beige*” adipocytes. Our data suggest that adiponectin may regulate thermogenesis in brown adipocytes via the following 4 independent and complementary cellular/molecular mechanisms: 1) Ablation of adiponectin decreases β3-AR expression, which directly inhibits UCP-1 gene expression and activity. 2) Ablation of adiponectin inhibits insulin signaling, which may reduce UCP-1 activity. 3) Ablation of adiponectin suppresses the signaling pathway of AMPK-SIRT1, which may in turn result in reduced mitochondrial biogenesis. 4) Ablation of adiponectin impairs mitochondrial dynamics in BAT, which may contribute to thermogenic dysfunction. Collectively, adiponectin ablation diminishes thermogenic activation by decreasing adrenergic activation, and impairing mitochondrial biosynthesis and/or dynamics, thus suppressing thermogenesis in *brown* and “*beige*” adipocytes

It has been reported that adiponectin activates AMPK-SIRT1 to regulate mitochondrial biogenesis and insulin sensitivity in muscle [49]. Similarly, it has been suggested that adiponectin, via SIRT1 and AMPK, regulates lipid metabolism in liver [50]. It has been reported that adipocyte AMPK is required for acute BAT-mediated thermogenesis [63], and that there is cross-talk between insulin signaling and AMPK signaling pathway [64]. AKT and AMPK activity is activated by cold exposure in BAT, and the activation of insulin signaling and AMPK are linked to enhanced UCP1 activity and thermogenic activation [65, 66]. Indeed, we found that adiponectin ablation suppresses AMPK activity in adipose tissues, and reduces expression of SIRT1 in BAT and subcutaneous fat in *Adipoq* ^−/−^ mice. Our data reveal that adiponectin ablation suppresses the AMPK-SIRT1 pathway, which may subsequently reduce mitochondrial biogenesis and suppress thermogenic function. Thus it is likely that adiponectin regulates thermogenic activity in BAT through different mechanisms: suppressing adrenergic activation of β3-AR, inhibiting insulin signaling, and/or deactivating the AMPK-SIRT1 pathway (Fig. 6).

## Conclusions

Our data demonstrate that adiponectin regulates thermogenesis in BAT and subcutaneous fat under cold exposure. Adiponectin-associated thermogenesis is not essential under normal housing temperature and *ad.lib* feeding condition, but is required for fasting and cold-challenged conditions. Adiponectin is required for maintaining body temperature in cold environment. Moreover, our data reveals that adiponectin ablation attenuates thermogenic signaling, reduces mitochondrial biogenesis and impairs mitochondrial dynamics, possibly by suppressing insulin signaling and the AMPK-SIRT1 pathway in BAT. Therefore, adiponectin is an important thermogenic regulator under energy deficit and cold environment, and adiponectin may serve as an effective therapeutic agent for hypothermia.

## Additional file

Additional file 1: Figure S1. Adiponectin ablation reduces UCP1 and IRS-1 protein levels. (PDF 65 kb)

## Abbreviations

*Adipoq*^−/−^: Adiponectin-null mice
AMPK: AMP-activated protein kinase
BAT: Brown adipose tissue
Drp1: Dynamin-related protein 1
Fis1: Fission 1 protein
Glut4: Glucose uptake regulator
GTT: Glucose tolerance tests
IR: Insulin receptor
IRS-1: Insulin receptor substrate 1
ITT: Insulin tolerance tests
Mfns: Mitofusins
OPA1: Optic atrophy gene 1
RMR: Resting metabolic rate
SIRT1: Sirtuin 1
SNS: Sympathetic nervous system
UCP-1: Uncoupling protein 1
UCP-2: Fat utilization regulator
WAT: Subcutaneous white adipose tissue
β3-AR: β3-adrenergic receptor
