# Supplementary material for: Adiponectin is required for maintaining normal body temperature in a cold environment
Source: BMC Physiol. 2017 Oct 23;17:8. doi: 10.1186/s12899-017-0034-7 (PMC5651620; doi:10.1186/s12899-017-0034-7)
Supplement: Additional file 1: Figure S1. — Adiponectin ablation reduces UCP1 and IRS-1 protein levels. (PDF 65 kb) [file 12899_2017_34_MOESM1_ESM.pdf]

Supplemental Fig. 1

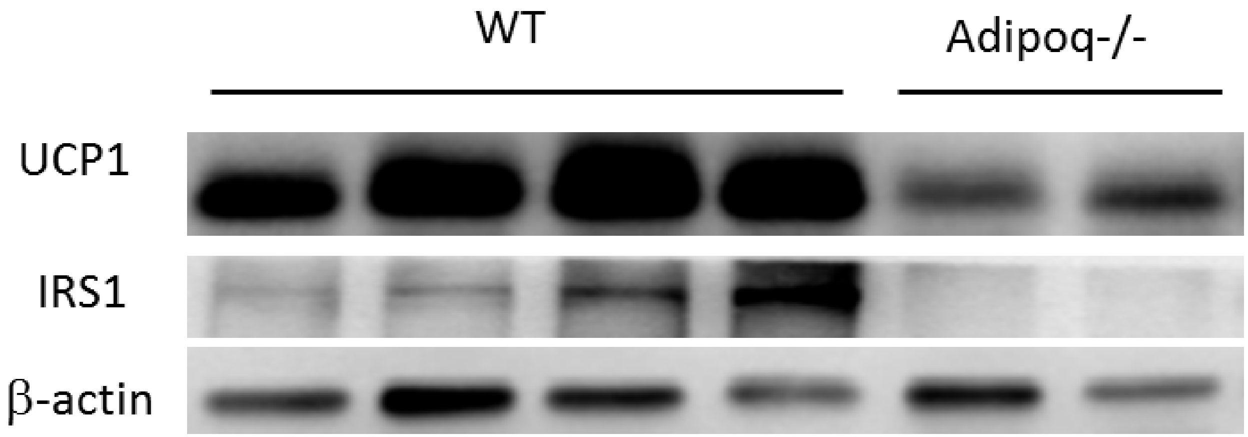

Supplemental Fig. 1. Adiponectin ablation reduces IRS-1 and UCP1 protein. BAT from 10-month old WT and *Adipoq*$^{-/-}$ mice was collected immediately following a 6 h cold (4°C) exposure. Western blot detection of IRS1 and UCP1.
